# Interaction Potential between a Uniformly Charged Square Nanoplate and Coplanar Nanowire

**DOI:** 10.3390/nano13232988

**Published:** 2023-11-21

**Authors:** Orion Ciftja

**Affiliations:** Department of Physics, Prairie View A&M University, Prairie View, TX 77446, USA; ogciftja@pvamu.edu

**Keywords:** potential, charge, square nanoplate, nanowire, quantum Hall effect, 73.20.Dx, 73.43.-f, 73.43.Cd

## Abstract

We study a structure consisting of two electrostatically interacting objects, a uniformly charged square nanoplate and a uniformly charged nanowire. A straightforward motivation behind this work is to introduce a model that allows a classical description of a finite two-dimensional quantum Hall system of few electrons when the Landau gauge is imposed. In this scenario, the uniformly charged square nanoplate would stand for the neutralizing background of the system while a uniformly charged nanowire would represent the resulting quantum striped state of the electrons. A second important feature of this model is that it also applies to hybrid charged nanoplate-nanowire systems in which the dominant interaction has electrostatic origin. An exact analytical expression for the electrostatic interaction potential between the uniformly charged square nanoplate and coplanar nanowire is obtained by using a special mathematical method adept for this geometry. It is found that the resulting interaction potential is finite, monotonic and slowly-varying for all locations of the nanowire inside the nanoplate.

## 1. Introduction

Two-dimensional (2D) systems such as infinitely thin layers or thin films [1,2,3,4] have attracted research interest time and time again due to their unique properties typically associated with a lower dimension as well as the predominance of quantum behavoir. The peculiar behavior of 2D materials potentially lead to various important applications in industry and have been the focus of many studies [5,6,7,8,9,10,11,12,13]. Current experimental techniques allow one to further reduce the dimensionality of space and fabricate one-dimensional (1D) systems which can be seen as thin wires [14].

The two-dimensional electron gas (2DEG) is a very prominent example of a low-dimensional system that continues to draw a lot of interest in condensed matter physics research and related fields [15]. Especially, 2D systems of electrons formed in a GaAs-AlGaAs heterostructure in presence of a strong perpendicular magnetic field show strange quantum behavoir which is peculiar and is associated with the so-called quantum Hall state of matter. The discovery of the integer quantum Hall effect [16] and fractional quantum Hall effect [17,18,19,20] is strong evidence in this regard.

The theoretical basis to understand quantum Hall phenomena is the quantum problem of a charged particle (an electron) confined to 2D space in a constant perpendicular magnetic field. Such a problem was first solved by Landau where the so-called Landau gauge for the magnetic field (as currently known) was also introduced [21]. The wave functions for the electrons in the Landau gauge look like stripes. They are extended in one direction, but they are exponentially localised around a given set of centers in the other perpendicular direction. The localization length is of the order of the magnetic length of the electrons, $l_{B}=\sqrt{\hbar /(|e|\;B)}$ where *ℏ* is the reduced Planck’s constant, |e| is the magnitude of the charge of the electron (same as charge of a proton) and $B=|\vec{B}|$ is the magnitude of the magnetic field perpendicular to the 2D plane of motion of electrons. For a magnetic field of B=10T, the magnetic length for an electron is $l_{B}\approx 8.1\times 10^{-9}\;$ m (thus, in the order of nanometers). Let us envision a situation in which we are dealing with a finite system consisting of a few electrons. In order to complete the theoretical model one must assume that the electrons move in presence of a positive neutralizing background charge that is uniformly distributed in 2D space. In the Landau gauge, the region that contains the neutralizing background charge may be chosen as a 2D square domain, but should not be chosen as a circular disk [22,23] which would represent the standard selection for the case of a symmetric gauge [24]. For small finite systems of *N* electrons the length, *L* of the square region is calculated to be $L=\sqrt{2\;\pi \;N/\nu }\;l_{B}$ where ν is the filling factor of a Landau level (an integer or fractional number). Therefore, for small given finite *N* and ν of the similar order of magnitude, one might visualize situations that lead to a square length, *L* of the order of several $l_{B}$. These rough estimates just serve the purpose to argue that for such conditions one may view the striped state of the electrons as a thin 1D nanowire with a length that is the same as the length of the square nanoplate. Furthermore, one may even argue that the stripes are relatively well separated from each other. Based on such a description, one can classically view such a striped state as a uniformly charged nanowire with a constant linear charge density, λ=q/L where *q* is the charge of the nanowire. Similarly, one can model the square neutralizing background as as a uniformly charged square nanoplate with a constant surface charge density, $\sigma =Q/L^{2}$ where *Q* is the charge of the nanoplate. More explicitly, if one looks at *N* electrons in presence of a 2D square jellium neutralizing background, one has q=−|e|<0 and Q=N|e|>0. In this manner, the overall net charge of the whole system is zero.

Charged nanoplates made of different types of materials are also commonly used as electrodes in the manufacturing of nanocapacitors [25,26]. Therefore, this model is important to describe the interaction of already synthesized nanosystems consisting of a charged nanoplate in presence of charged 1D nanowires. For example, monoatomic linear carbon 1D nanowires have been produced in laboratory settings. These structures have a great potential to be used as nanojunctions/nanoconductors for novel electronic devices. Materials such as 2D graphene sheets are also often used as typical plate electrodes in nanoelectronic devices consisting of many elements including a variety of 1D wires that act as nanojunctions. It is known that some of the electric properties of 1D nanojunctions are controlled by quantum effects, for instance, the conductance of silicon-doped carbon wire nanojunctions [27]. However, there are instances where classical electric effects dominate. For example, the anomalous size-dependent nanocapacitance of a boron nitride-graphene nanocapacitor was initially thought to be due to quantum effects [28], but later on, the behavior was explained accurately by appealing to classical electrostatic phenomena [29]. This means that it is worthy to investigate the nature of the electrostatic interaction for the system that comprises a uniformly charged nanoplate and a uniformly charged nanowire that is coplanar with it.

The article is organized as follows. In Section 2 we explain the theory and the model of the system under consideration. In Section 3 we discuss and explain the main results of this work. In Section 4 we summarize key findings and provide a few concluding remarks.

## 2. Theory and Model

**Figure 1:**
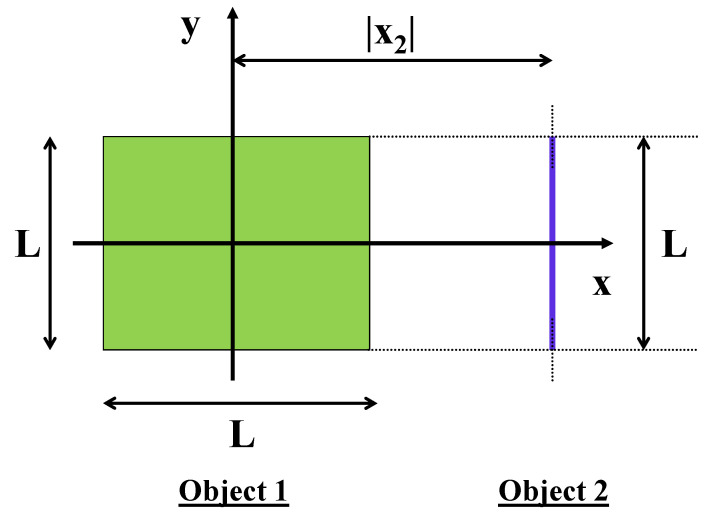
Schematic view of a system consisting of a uniformly charged nanoplate (object 1, green) and a uniformly charged nanowire (object 2, blue). The two objects are coplanar and lie on the x−y plane. The origin of the system of coordinates is chosen at the center of the square nanoplate with *x* and *y* axes parallel to its edges. The square nanoplate has an arbitrary length, *L*. The nanowire has an identical length and is positioned parallel to the edge of the square nanoplate (along the *y* direction) and opposite to it. The square nanoplate has a uniform surface charge density, $\sigma =Q/L^{2}$ where *Q* is the total charge uniformly spread over the area of the square nanoplate. The nanowire has a uniform linear charge density, λ=q/L where *q* is the total charge uniformly spread over the length of the nanowire. The distance from the center of the square nanoplate to the center of the nanowire is $|x_{2}|$.

The model under consideration consists of a uniformly charged square nanoplate (object 1) and a uniformly charged nanowire (object 2). The two objects are on the same plane, namely, they are coplanar. The square nanoplate has an arbitrary length, *L*. The nanowire has the same length, *L* as the square nanoplate. The nanowire is parallel and opposite to the edge of the square nanoplate at an arbitrary separation distance. The positioning of the nanowire relative to the square nanoplate is shown in Figure 1. Quite generally, we assume that each of the two objects has an arbitrary net charge. It is assumed that the square nanoplate has a net charge, *Q* while the nanowire’s total charge is *q*. For the square nanoplate:

(1) $$\sigma =\frac{Q}{L^{2}}\;.$$

Similarly, for the nanowire:

(2) $$\lambda =\frac{q}{L}\;.$$

We choose a 2D Cartesian system of coordinates with origin at the center of the square nanoplate. The *x* and *y* axes of the system of coordinates are oriented as shown in Figure 1. We associate coordinates, ${\vec{r}}_{1}=\left(x_{1},y_{1}\right)$ with object 1 (square nanoplate) and ${\vec{r}}_{2}=\left(x_{2},y_{2}\right)$ with object 2 (nanowire). The 2D domain of the square nanoplate variables $x_{1}$ and $y_{1}$ is:

(3) $$D_{1}:\left\{\begin{array}{c} -\frac{L}{2}\leq x_{1}\leq +\frac{L}{2}\\ -\frac{L}{2}\leq y_{1}\leq +\frac{L}{2}\;. \end{array}\right.$$

Correspondingly, the domain of the nanowire variables $x_{2}$ and $y_{2}$ is:

(4) $$D_{2}:\left\{\begin{array}{c} x_{2}=arbitrary\\ -\frac{L}{2}\leq y_{2}\leq +\frac{L}{2}\;. \end{array}\right.$$

This means that the separation distance between the center of the square nanoplate and the center of the nanowire is $|x_{2}|$.

We consider the elementary charges, $dQ=\sigma \;d^{2}r_{1}$ (object 1, square nanoplate) and $dq=\lambda \;dy_{2}$ (object 2, nanowire) where $d^{2}r_{1}=dx_{1}dy_{1}$ is an elementary surface area on the square nanoplate at ${\vec{r}}_{1}=\left(x_{1},y_{1}\right)$ while $dy_{2}$ is an elementary length in the nanowire located at ${\vec{r}}_{2}=\left(x_{2},y_{2}\right)$. We denote the interaction potential between the two charged objects as $U_{12}\left(x_{2}\right)$ since it can be seen that it depends only on the variable, $x_{2}$. We will show at a later stage that such an interaction potential depends on the quantity $|x_{2}|$, a conclusion that makes perfect sense based on symmetry arguments. The electrostatic interaction potential energy of the structure is calculated from:

(5) $$U_{12}\left(x_{2}\right)=k\;\sigma \;\lambda \underset{D_{1}}{\;\int \int \;}dx_{1}\;dy_{1}\int_{D_{2}}dy_{2}\;\;\frac{1}{|{\vec{r}}_{1}-{\vec{r}}_{2}|}\;,$$

where *k* is the Coulomb’s electric constant, ${\vec{r}}_{i}=\left(x_{i},y_{i}\right)$(i=1,2) are 2D vectors, the 2D integral is over the square nanoplate domain $D_{1}$ and the line integral is over the nanowire domain $D_{2}$. The calculation of the integral in Equation (5) is practically impossible to be dealt with via direct integration. However, a special mathematical approach adept for rectangular geometry enables us to facilitate the calculations. As a result, in spite of the challenges, we are able to obtain an exact analytical result for the interaction potential for the case under consideration.

## 3. Results and Discussions

Our method to calculate the integral in Equation (5), starts with the introduction of a Laplace-like transformation of the quantity $1/|{\vec{r}}_{1}-{\vec{r}}_{2}|$ as follows:

(6) $$\frac{1}{|{\vec{r}}_{1}-{\vec{r}}_{2}|}=\frac{2}{\sqrt{\pi }}\int_{0}^{\infty }du\;e^{-u^{2}\;{\left({\vec{r}}_{1}-{\vec{r}}_{2}\right)}^{2}}=\frac{2}{\sqrt{\pi }}\int_{0}^{\infty }du\;e^{-u^{2}\;{\left(x_{1}-x_{2}\right)}^{2}}\;e^{-u^{2}\;{\left(y_{1}-y_{2}\right)}^{2}}\;.$$

We substitute the result from Equation (6) into Equation (5) and write:

(7) $$U_{12}\left(x_{2}\right)=k\;\sigma \;\lambda \;\frac{2}{\sqrt{\pi }}\int_{0}^{\infty }du\;\int_{-\frac{L}{2}}^{+\frac{L}{2}}dx_{1}\;\;e^{-u^{2}\;{\left(x_{1}-x_{2}\right)}^{2}}\int_{-\frac{L}{2}}^{+\frac{L}{2}}dy_{1}\;\int_{-\frac{L}{2}}^{+\frac{L}{2}}dy_{2}\;\;e^{-u^{2}\;{\left(y_{1}-y_{2}\right)}^{2}}\;.$$

Now, we define two auxiliary functions which have the following form:

(8) $$f\left(u,x_{2},L\right)=\int_{-\frac{L}{2}}^{+\frac{L}{2}}dx_{1}\;e^{-u^{2}\;{\left(x_{1}-x_{2}\right)}^{2}}\;,$$

and

(9) $$f\left(u,y_{2},L\right)=\int_{-\frac{L}{2}}^{+\frac{L}{2}}dy_{1}\;e^{-u^{2}\;{\left(y_{1}-y_{2}\right)}^{2}}\;.$$

These functions, are explicitly calculated in Appendix A and read:

(10) $$f\left(u,x_{2},L\right)=\frac{\sqrt{\pi }}{2\;u}\left\{er\;f\left[u\;\left(\frac{L}{2}+x_{2}\right)\right]+er\;f\left[u\;\left(\frac{L}{2}-x_{2}\right)\right]\right\}\;,$$

(11) $$f\left(u,y_{2},L\right)=\frac{\sqrt{\pi }}{2\;u}\left\{er\;f\left[u\;\left(\frac{L}{2}+y_{2}\right)\right]+er\;f\left[u\;\left(\frac{L}{2}-y_{2}\right)\right]\right\}\;,$$

where

(12) $$er\;f\left(x\right)=\frac{2}{\sqrt{\pi }}\;\int_{0}^{x}e^{-t^{2}}\;dt\;,$$

is an error function.

By relying on the definitions from Equations (8) and (9), one writes the expression in Equation (7) in a succint way as:

(13) $$U_{12}\left(x_{2}\right)=k\;\sigma \;\lambda \;\frac{2}{\sqrt{\pi }}\int_{0}^{\infty }du\;f\left(u,x_{2},L\right)\;\int_{-\frac{L}{2}}^{+\frac{L}{2}}dy_{2}\;f\left(u,y_{2},L\right)\;.$$

The resulting integral, $\int_{-\frac{L}{2}}^{+\frac{L}{2}}dy_{2}\;f\left(u,y_{2},L\right)$ in Equation (13) can be calculated exactly as shown in Appendix B. The final result is:

(14) $$\int_{-\frac{L}{2}}^{+\frac{L}{2}}dy_{2}\;f\left(u,y_{2},L\right)=\frac{\sqrt{\pi }\;\;\left(u\;L\right)\;\;erf\left(u\;L\right)+e^{-{\left(u\;L\right)}^{2}}-1}{u^{2}}\;.$$

By using the results from Equation (10) and from Equation (14) one can write the interaction potential in Equation (13) as:

(15) $$U_{12}\left(x_{2}\right)=k\;\sigma \;\lambda \;\int_{0}^{\infty }\frac{du}{u}\;\left\{erf\left[u\;\left(\frac{L}{2}+x_{2}\right)\right]+erf\left[u\;\left(\frac{L}{2}-x_{2}\right)\right]\right\}\;\frac{\sqrt{\pi }\;\;\left(u\;L\right)\;\;erf\left(u\;L\right)+e^{-{\left(u\;L\right)}^{2}}-1}{u^{2}}\;.$$

One can easily check that:

(16) $$\sigma \;\lambda =\frac{Q\;q}{L}\;\;\frac{1}{L^{2}}\;.$$

This means that the quantity in Equation (15) can be written as:

(17) $$U_{12}\left(x_{2}\right)=\frac{k\;Q\;q}{L}\;\int_{0}^{\infty }\frac{du}{u}\;\left\{er\;f\left[u\;\left(\frac{L}{2}+x_{2}\right)\right]+er\;f\left[u\;\left(\frac{L}{2}-x_{2}\right)\right]\right\}\;\frac{\sqrt{\pi }\;\;\left(u\;L\right)\;\;er\;f\left(u\;L\right)+e^{-{\left(u\;L\right)}^{2}}-1}{{\left(u\;L\right)}^{2}}\;.$$

At this juncture, let us introduce another auxiliary function that has the following form:

(18) $$g\left(t\right)=\sqrt{\pi }\;\;\;\frac{er\;f(t)}{t}+\frac{e^{-t^{2}}-1}{t^{2}}\;.$$

This enables us to write the result in Equation (17) as:

(19) $$U_{12}\left(x_{2}\right)=\frac{k\;Q\;q}{L}\;\int_{0}^{\infty }\frac{du}{u}\;\left\{er\;f\left[u\;\left(\frac{L}{2}+x_{2}\right)\right]+er\;f\left[u\;\left(\frac{L}{2}-x_{2}\right)\right]\right\}\;g\left(u\;L\right)\;.$$

One can immediately note by checking the expression in Equation (19) that the interaction potential is an even function of the variable $x_{2}$, namely:

(20) $$U_{12}\left(x_{2}\right)=U_{12}\left(-x_{2}\right)\;.$$

Therefore, it is perfectly legitimate to replace $x_{2}$ with $|x_{2}|$ everywhere it appears:

(21) $$U_{12}\left(x_{2}\right)=\frac{k\;Q\;q}{L}\;\int_{0}^{\infty }\frac{du}{u}\;\left\{er\;f\left[u\;\left(\frac{L}{2}+\left|x_{2}\right|\right)\right]+er\;f\left[u\;\left(\frac{L}{2}-\left|x_{2}\right|\right)\right]\right\}\;g\left(u\;L\right)\;.$$

The expression in Equation (21) is valid for arbitrary *L* and $x_{2}$. The case L=0 would lead to a Coulomb expression of the form, $U_{12}\left(x_{2}\right)=k\;Q\;q/\left|x_{2}\right|$. To obtain this result one must exercise some care while handling the L→0 limit. Having said that, from now on, we assume L≠0 and proceed to introduce two new dimensionless variables of the form:

(22) $$t=u\;L\;\;\;;\;\;\;r=\frac{|x_{2}|}{L}\geq 0\;.$$

This approach allows us to rewrite the quantity in Equation (21) as:

(23) $$U_{12}\left(r\right)=\frac{k\;Q\;q}{L}\;\int_{0}^{\infty }\frac{dt}{t}\;\left\{erf\left[t\;\left(\frac{1}{2}+r\right)\right]+erf\left[t\;\left(\frac{1}{2}-r\right)\right]\right\}\;g\left(t\right)\;,$$

where function, g(t) is given from Equation (18) and $r=|x_{2}|/L\geq 0$ is the center-to-center distance between the two objects expressed in units of *L*. Note that, at this point, we changed the argument of the function in Equation (23) from variable $x_{2}$ to variable *r*. The rationale of this choice of notation is to show explicitly the dependence of the interaction potential on the dimensionless distance, $r=|x_{2}|/L$. By using the expression for g(t) in Equation (18), one writes $U_{12}\left(r\right)$ as:

(24) $$\begin{array}{ccc} U_{12}\left(r\right) & = & \frac{k\;Q\;q}{L}\;\left\{\sqrt{\pi }\;\int_{0}^{\infty }\frac{dt}{t^{2}}\;er\;f\left[t\left(\frac{1}{2}+r\right)\right]\;er\;f\left(t\right)+\int_{0}^{\infty }\frac{dt}{t^{3}}\;er\;f\left[t\left(\frac{1}{2}+r\right)\right]\;\left(e^{-t^{2}}-1\right)+\right.\\ & \; & \left.\sqrt{\pi }\;\int_{0}^{\infty }\frac{dt}{t^{2}}\;er\;f\left[t\left(\frac{1}{2}-r\right)\right]\;er\;f\left(t\right)+\int_{0}^{\infty }\frac{dt}{t^{3}}\;er\;f\left[t\left(\frac{1}{2}-r\right)\right]\;\left(e^{-t^{2}}-1\right)\right\}\;. \end{array}$$

The exact calculation of the integral expressions in Equation (24) depends on the calculation of two integrals that read:

(25) $$\int_{0}^{\infty }dx\;\frac{er\;f(a\;x)\;er\;f(x)}{x^{2}}\;,$$

and

(26) $$\int_{0}^{\infty }\frac{dx}{x^{3}}\;er\;f\left(a\;x\right)\;\left(e^{-x^{2}}-1\right)\;,$$

where *a* is a real constant. The integral in Equation (25) is calculated in Appendix C and the final result is:

(27) $$\int_{0}^{\infty }dx\;\frac{er\;f(a\;x)\;er\;f(x)}{x^{2}}=\frac{2}{\sqrt{\pi }}\;\left[a\;\sinh^{-1}\left(\frac{1}{\sqrt{a^{2}}}\right)+\sinh^{-1}\left(a\right)\right]\;,$$

where $\sinh^{-1}\left(x\right)$ is an inverse hyperbolic sine function:

(28) $$\sinh^{-1}\left(x\right)=\ln\left(x+\sqrt{x^{2}+1}\right)\;.$$

The integral in Equation (26) is calculated in Appendix D and the final result is:

(29) $$\int_{0}^{\infty }\frac{dx}{x^{3}}\;er\;f\left(a\;x\right)\;\left(e^{-x^{2}}-1\right)=a\;\left(\sqrt{a^{2}}-\sqrt{a^{2}+1}\right)-\sinh^{-1}\left(a\right)\;.$$

At this point we introduce the following function:

(30) $$F\left(a\right)=\sqrt{\pi }\;\int_{0}^{\infty }dx\;\frac{er\;f(a\;x)\;er\;f(x)}{x^{2}}+\int_{0}^{\infty }\frac{dx}{x^{3}}\;er\;f\left(a\;x\right)\;\left(e^{-x^{2}}-1\right)\;.$$

From the results in Equations (27) and (29), one can verify that this function is:

(31) $$F\left(a\right)=2\;a\;\;\sinh^{-1}\left(\frac{1}{\sqrt{a^{2}}}\right)+\sinh^{-1}\left(a\right)+a\;\left(\sqrt{a^{2}}-\sqrt{a^{2}+1}\right)\;.$$

The final step is to look at the expression for the interaction potential in Equation (24) and recognize that one can write this quantity very succintly in a compact form as:

(32) $$U_{12}\left(r\right)=\frac{k\;Q\;q}{L}\;\left[F\left(\frac{1}{2}+r\right)+F\left(\frac{1}{2}-r\right)\right]\;,$$

where function F(a) is given from Equation (31). The expression in Equation (32) is the final exact analytical result for the electrostatic interaction potential between a uniformly charged square nanoplate with arbitrary length, *L* and a uniformly charged nanowire with the same length for the geometric setup shown in Figure 1 at a given dimensionless center-to-center separation distance, $r=|x_{2}|/L$. A special situation arises when the nanowire is exactly at the center of the square nanoplate. For such a case one can calculate that:

(33) $$U_{12}\left(r=0\right)=\frac{k\;Q\;q}{L}\;\left[2\;\sinh^{-1}\left(2\right)+2\;\sinh^{-1}\left(\frac{1}{2}\right)+\frac{1}{2}\left(1-\sqrt{5}\right)\right]\approx 3.23166\;\;\frac{k\;Q\;q}{L}\;.$$

**Figure 2:**
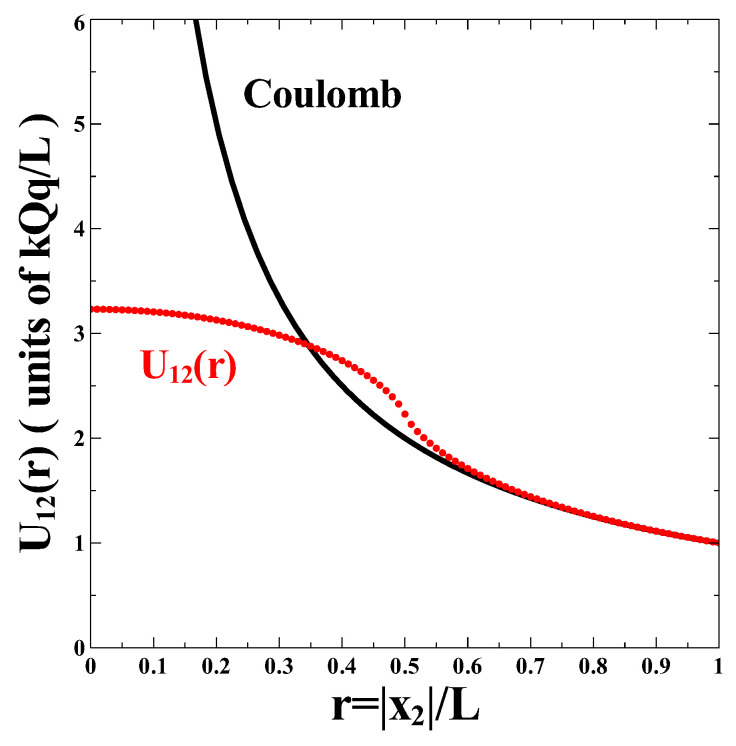
Interaction potential, $U_{12}\left(r\right)$ between a uniformly charged square nanoplate with length, *L* and total charge *Q* and a uniformly charged nanowire with identical length and total charge *q*. The quantity is calculated as a function of $r=|x_{2}|/L$ where $|x_{2}|$ is the center-to-center separation distance between the two objects (filled red circles) for the configuration shown in Figure 1. The result is compared to the case of a standard Coulomb interaction potential, $U_{C}\left(r\right)=k\;Q\;q/r$ (solid line). The interaction potential is expressed in units of kQq/L.

In Figure 2 we plot the interaction potential, $U_{12}\left(r\right)$ as a function of $r=|x_{2}|/L$ The interaction potential is given in units of kQq/L. The result obtained is compared to the case of a standard Coulomb interaction potential (solid line) of the form:

(34) $$U_{C}\left(r\right)=\frac{1}{r/L}\;\frac{k\;Q\;q}{L}\;.$$

The two functions differ in a substantial way only for $r=|x_{2}|/L<1/2$. This means that the interaction potential $U_{12}\left(r\right)$ is approximately Coulomb as long as the nanowire is separated in space from the nanoplate. However, the interaction potential is strikingly non-Coulomb when the nanowire touches the nanoplate and moves towards its center. One exception is the critical distance, $r_{c}\approx 0.35$ where the two curves intersect. One can see that $U_{C}\left(r\right)$ is weaker than $U_{12}\left(r\right)$ for distances up to this intersection point. For distances smaller than this intersection point, the Coulomb potential $U_{C}\left(r\right)$ grows very fast as it diverges in the r→0 limit. On the other hand, the interaction potential $U_{12}\left(r\right)$ more or less plateaus and eventually reaches a finite value at r=0 as calculated in Equation (33). In order to understand better this behavior, we calculated the difference between the Coulomb interaction potential and the resulting potential, $U_{12}\left(r\right)$:

(35) $$\Delta U\left(r\right)=U_{C}\left(r\right)-U_{12}\left(r\right)\;.$$

**Figure 3:**
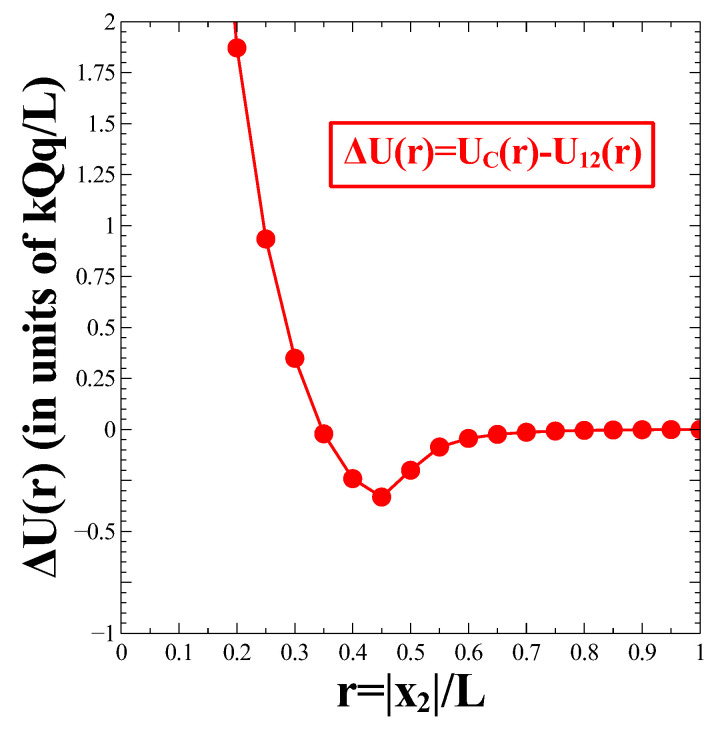
Potential difference, $\Delta U\left(r\right)=U_{C}\left(r\right)-U_{12}\left(r\right)$ as a function of $r=|x_{2}|/L$. The filled red circles represent data points while the solid red line is a guide for the eyes. The interaction potential is expressed in units of kQq/L.

The result is shown in Figure 3 where we display ΔU(r) (in units of kQq/L) as a function of the dimensionless distance, $r=|x_{2}|/L$. The resulting ΔU(r) is non-negative for up to a critical distance, $r_{c}\approx 0.35$ and becomes negative for larger values of *r*. One can observe that the function ΔU(r) eventually reaches a minimum at some distance larger than $r_{c}$. These features, in broad terms, are reminiscent of a Lennard-Jones (LJ) potential.

**Figure 4:**
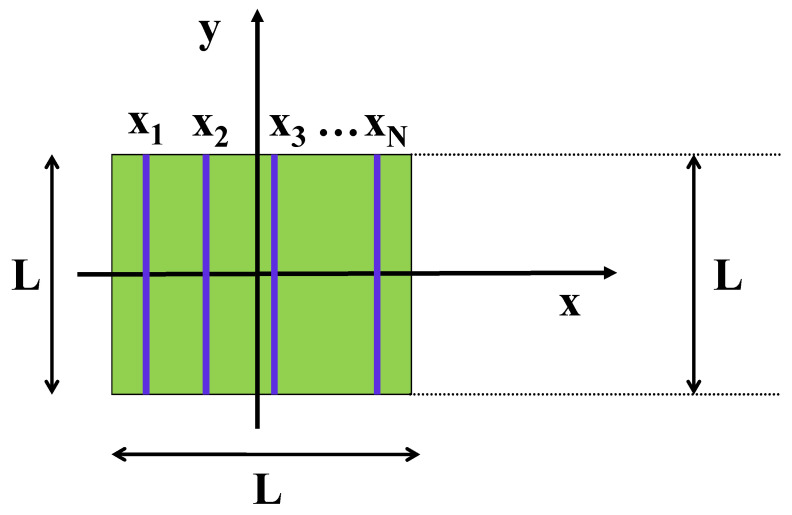
Schematic view of a system of *N* uniformly charged nanowires (blue lines) contained within the area of a uniformly charged square nanoplate (green square).

Obtaining the interaction potential between a charged nanoplate-nanowire coplanar system as considered in this work is only the first step towards studying a more general situation in which an arbitrary number of N≥2 uniformly charged nanowires interact with a larger uniformly charged nanoplate. Such a more general system is schematically shown in Figure 4.

Assume that, for the given geometric arrangement, the *i*-th nanowire is located at position $r_{i}=\left|x_{i}\right|/L$ where $r_{i}$ represents the separation distance between the center of the square nanoplate and the center of the *i*-th nanowire (i=1,2,…,N). The total energy of the system to be calculated may be written as:

(36) $$U\left(N\right)=\sum_{i=1}^{N}U_{12}\left(r_{i}\right)+\sum_{i<j}^{N}U^{\;\prime }\left(\right|x_{i}-x_{j}\left|\right)\;,$$

where the potential $U_{12}\left(r_{i}\right)$ is given from Equation (32) while $U^{\;\prime }\left(\right|x_{i}-x_{j}\left|\right)$ represents the nanowire-nanowire interaction energy for the arrangement of two identical parallel nanowires located, respectively, at position $x_{i}$ and $x_{j}$. The self-energy, namely, the stored electrostatic energy due to the uniformly charged nanoplate is a mere constant and, thus, it is not included in the expression in Equation (36). Knowing the exact form of the interaction potentials such as $U_{12}\left(r_{i}\right)$ in the present case, will surely improve the efficiency of simulation methods. The calculation of the total energy in Equation (36) can be done only numerically when one considers systems with a large *N*. The outcome of such a numerical computation is essential for the identification of the most stable configuration of the system, namely, the specific one with the lowest energy possible.

## 4. Conclusions

To summarize, in this work we calculated exactly, in analytic form, the interaction potential between a uniformly charged square nanoplate and a uniformly charged nanowire for a given spatial configuration of the two objects as shown schematically in Figure 1. The exact result was obtained with help from a special method that relies on a suitable Laplace-like transformation of variables. This approach allows one to streamline and facilitate the calculation of the multi-dimensional integrals that appear in the expression of the interaction potential in Equation (5). Multi-dimensional integrals as encountered in this work are very cumbersome to be dealt with by mainstream standard integration methods. Such multi-dimensional integrals typically appear in the calculation of the electrostatic energy, namely, stored electrostatic energy of a uniformly charged object with square/cube geometry. Based on my knowledge, the only way to calculate exactly such multi-dimensional integrals is by using the mathematical transformation in Equation (6). This very useful transformation has allowed for an exact analytical calculation of the electrostatic self-energy (electrostatic energy stored) for very complicated objects such as a uniformly charged square plate [30], a uniformly charged cube [31] and, even, a uniformly charged rectangular plate with arbitrary length and width [32].

The final result that we derived in this work is conveniently and elegantly written in compact form in terms of an auxiliary function defined in Equation (31). This function depends on the center-to-center separation distance between the two objects conveniently expressed in units of *L* in order to make it dimensionless. As expected, the Coulomb expression for the interaction potential is recovered in the L→0 limit. For the more relevant L≠0 case, it was noted that the interaction potential is finite even when the centers of the two objects coincide. Structures with charged nanoplates and nanowires are regularly encountered within the framework of 2D nanoscale systems or metalic thin films in which the electrostatic interaction is one of the dominant factors [33,34]. The common assumption of uniform charge distribution for such objects is not only legitimate, but it is the only one that may lead to exact analytical results for particular cases. Therefore, the exact analytical expression derived in this work stands as important in its own merit.

In addition, this result is also useful for a classical treatment of small 2D quantum Hall systems electrons when the Landau gauge is used [35]. In this picture, the uniformly charged square nanoplate would represent the neutralizing jellium background of the system while the uniformly charged nanowire would represent the striped state of the electron that emerges in a Landau gauge. These are the conditions where quantum Hall behavior is encountered and a quantum treatment is required [36,37,38,39,40,41]. Nonetheless, it is logical to argue that an classical model of the nature considered in this work may work reasonably well as long as we are dealing with small systems of electrons that obviously are confined to a small 2D domain with dimensions in the nanoscale (though with *L* markedly larger than $l_{B}$ which represents the width of the striped state). For such conditions, the nanoplate and nanowire model makes sense and seems worthy of investigation. Another system where this model may have some bearing is that of the charge-density wave states of electrons [42]. As a final remark, we also point out that the exact analytical results that we derived can be helpful to assess the accuracy of standard numerical methods which are typically used to carry out multi-dimensional integral calculations for cumbersome systems such as those without circular symmetry.

## Data Availability

The data presented in this study are available upon request from the author.

## Appendix A. Functions, *f*(*u*, *x*_2_, *L*) and *f*(*u*, *y*_2_, *L*)

Function $f(u,x_{2},L)$ is given in integral form as:

(A1) $$f\left(u,x_{2},L\right)=\int_{-\frac{L}{2}}^{+\frac{L}{2}}dx_{1}\;e^{-u^{2}\;{\left(x_{1}-x_{2}\right)}^{2}}\;.$$

We denote $v=x_{1}-x_{2}$. The expression in Equation (A1) is rewritten in terms of this new variable as:

(A2) $$f\left(u,x_{2},L\right)=\int_{-\frac{L}{2}-x_{2}}^{+\frac{L}{2}-x_{2}}dv\;e^{-u^{2}\;v^{2}}\;.$$

At this juncture, we apply the ensuing integral formula:

(A3) $$\int dx\;e^{-a^{2}\;x^{2}}=\frac{\sqrt{\pi }}{2\;a}\;er\;f\left(a\;x\right)\;,$$

where $er\;f\left(x\right)=2/\sqrt{\pi }\;\int_{0}^{x}e^{-t^{2}}\;dt$ is an error function and *a* is a real constant. With help from such an integral formula, one obtains:

(A4) $$f\left(u,x_{2},L\right)=\frac{\sqrt{\pi }}{2\;u}\left\{er\;f\left[u\;\left(\frac{L}{2}-x_{2}\right)\right]-er\;f\left[u\;\left(-\frac{L}{2}-x_{2}\right)\right]\right\}\;.$$

The error function is an odd function, namely, erf(−x)=−erf(x). This allows us to rewrite the quantity in Equation (A4) as:

(A5) $$f\left(u,x_{2},L\right)=\frac{\sqrt{\pi }}{2\;u}\left\{er\;f\left[u\;\left(\frac{L}{2}+x_{2}\right)\right]+er\;f\left[u\;\left(\frac{L}{2}-x_{2}\right)\right]\right\}\;.$$

The other function $f(u,y_{2},L)$ given in integral form is:

(A6) $$f\left(u,y_{2},L\right)=\int_{-\frac{L}{2}}^{+\frac{L}{2}}dy_{1}\;e^{-u^{2}\;{\left(y_{1}-y_{2}\right)}^{2}}\;.$$

The calculation of the integral appearing in Equation (A6) follows the same approach as that for the $f(u,x_{2},L)$ case. The final result is:

(A7) $$f\left(u,y_{2},L\right)=\frac{\sqrt{\pi }}{2\;u}\left\{er\;f\left[u\;\left(\frac{L}{2}+y_{2}\right)\right]+er\;f\left[u\;\left(\frac{L}{2}-y_{2}\right)\right]\right\}\;.$$

## Appendix B. Calculation of the Integral, $\int_{-\frac{L}{2}}^{+\frac{L}{2}}$*dy*_2_ *f* (*u*, y_2_, *L*)

The integral to calculate is:

(A8) $$\int_{-\frac{L}{2}}^{+\frac{L}{2}}dy_{2}\;f\left(u,y_{2},L\right)\;,$$

where

(A9) $$f\left(u,y_{2},L\right)=\frac{\sqrt{\pi }}{2\;u}\left\{er\;f\left[u\;\left(\frac{L}{2}+y_{2}\right)\right]+er\;f\left[u\;\left(\frac{L}{2}-y_{2}\right)\right]\right\}\;.$$

We substitute the expression from Equation (A9) into Equation (A8):

(A10) $$\int_{-\frac{L}{2}}^{+\frac{L}{2}}dy_{2}\;f\left(u,y_{2},L\right)=\frac{\sqrt{\pi }}{2\;u}\;\left\{\int_{-\frac{L}{2}}^{+\frac{L}{2}}dy_{2}\;er\;f\left[u\;\left(\frac{L}{2}+y_{2}\right)\right]+\int_{-\frac{L}{2}}^{+\frac{L}{2}}dy_{2}\;er\;f\left[u\;\left(\frac{L}{2}-y_{2}\right)\right]\right\}\;.$$

The calculation of the first integral in Equation (A10) is simplified by inserting a dummy variable, $z=u\left(\frac{L}{2}+y_{2}\right)$. Similarly, one introduces the dummy variable, $z=u\left(\frac{L}{2}-y_{2}\right)$ for second integral in Equation (A10). As a result, one obtains:

(A11) $$\int_{-\frac{L}{2}}^{+\frac{L}{2}}dy_{2}\;f\left(u,y_{2},L\right)=\frac{\sqrt{\pi }}{u^{2}}\;\int_{0}^{u\;L}dz\;er\;f\left(z\right)\;.$$

The following integration formula applies:

(A12) $$\int dx\;er\;f\left(x\right)=x\;er\;f\left(x\right)+\frac{1}{\sqrt{\pi }}\;e^{-x^{2}}\;.$$

By applying the integral formula from Equation (A12) for the quantity in Equation (A11) one obtains:

(A13) $$\int_{-\frac{L}{2}}^{+\frac{L}{2}}dy_{2}\;f\left(u,y_{2},L\right)=\frac{\sqrt{\pi }\;\;\left(u\;L\right)\;\;er\;f\left(u\;L\right)+e^{-{\left(u\;L\right)}^{2}}-1}{u^{2}}\;.$$

## Appendix C. Calculation of the Integral $\int_{0}^{\infty }dx\frac{er\;f(ax)er\;f(x)}{x^{2}}$

The integral that we want to calculate is:

(A14) $$I\left(a\right)=\int_{0}^{\infty }dx\;\frac{er\;f(a\;x)\;er\;f(x)}{x^{2}}\;,$$

where *a* is a real constant. We apply integration by parts (∫udv=uv−∫vdu) where u=erf(ax)erf(x) and $dv=dx/x^{2}$. One has:

(A15) $$I\left(a\right)=\frac{2}{\sqrt{\pi }}\;\left[a\;\int_{0}^{\infty }dx\;e^{-a^{2}\;x^{2}}\;\frac{er\;f(x)}{x}+\int_{0}^{\infty }dx\;e^{-x^{2}}\;\frac{er\;f(a\;x)}{x}\right]\;.$$

We obtained the result in Equation (A15) after noticing that:

(A16) $$\lim_{x\to 0}\frac{er\;f(a\;x)\;er\;f(x)}{x}=\lim_{x\to \infty }\frac{er\;f(a\;x)\;er\;f(x)}{x}=0\;.$$

The first integral in the right-hand-side of Equation (A15) is calculated from:

(A17) $$\int_{0}^{\infty }dx\;e^{-a^{2}\;x^{2}}\;\frac{er\;f(x)}{x}=\sinh^{-1}\left(\frac{1}{\sqrt{a^{2}}}\right)\;,$$

where $\sinh^{-1}\left(x\right)=\ln\left(x+\sqrt{x^{2}+1}\right)$ is an inverse sine hyperbolic function. The second integral in the right-hand-side of Equation (A15) is:

(A18) $$\int_{0}^{\infty }dx\;\;e^{-x^{2}}\;\;\frac{er\;f(a\;x)}{x}=\sinh^{-1}\left(a\right)\;.$$

By combining the outcomes from Equations (A17) and (A18), one writes the quantity in Equation (A15) as:

(A19) $$I\left(a\right)=\frac{2}{\sqrt{\pi }}\;\left[a\;\sinh^{-1}\left(\frac{1}{\sqrt{a^{2}}}\right)+\sinh^{-1}\left(a\right)\right]\;.$$

One can look at Equation (A19) and verify that the expected result, I(−a)=−I(a) applies given that $\sinh^{-1}\left(x\right)$ is an odd function of *x*.

## Appendix D. Calculation of the Integral $\int_{0}^{\infty }\frac{dx}{x^{3}}\;er\;f\left(a\;x\right)\;\left(e^{-x^{2}}-1\right)$

The integral to calculate:

(A20) $$I\left(a\right)=\int_{0}^{\infty }\frac{dx}{x^{3}}\;er\;f\left(a\;x\right)\;\left(e^{-x^{2}}-1\right)\;,$$

where *a* is real and erf(x) is an error function. We write the quantity in Equation (A20) in the following form:

(A21) $$I\left(a\right)=I_{1}\left(a\right)-I_{2}\left(a\right)\;,$$

where

(A22) $$I_{1}\left(a\right)=\int_{0}^{\infty }\frac{dx}{x^{3}}\;e^{-x^{2}}\;er\;f\left(a\;x\right)\;,$$

and

(A23) $$I_{2}\left(a\right)=\int_{0}^{\infty }\frac{dx}{x^{3}}\;er\;f\left(a\;x\right)\;.$$

Basically, we wrote the original integral in Equation (A20) as a difference of two integrals, $I_{1}\left(a\right)$ and $I_{2}\left(a\right)$. At this juncture, we anticipate the result that each of these two integrals may contain singularities (infinities). Nonetheless, it will be found that all singularities disappear when the $I_{1}\left(a\right)-I_{2}\left(a\right)$ is calculated.

Firstly, let us calculate $I_{1}\left(a\right)$. We use integration by parts (∫udv=uv−∫vdu) where we denote $u=e^{-x^{2}}\;er\;f\left(a\;x\right)$ and $dv=dx/x^{3}$. We obtain the following result:

(A24) $$I_{1}\left(a\right)=-\frac{1}{2}\;\frac{e^{-x^{2}}\;er\;f\left(a\;x\right)}{x^{2}}{|}_{0}^{\infty }-\int_{0}^{\infty }\frac{dx}{x}\;e^{-x^{2}}\;er\;f\left(a\;x\right)+\frac{a}{\sqrt{\pi }}\;\int_{0}^{\infty }\frac{dx}{x^{2}}\;e^{-\left(a^{2}+1\right)\;x^{2}}\;.$$

Note that the limits of integration are from 0 to *∞*. This means that the first term in the right-hand-side of Equation (A24) diverges for x=0 (assuming that *a* is not zero) since:

(A25) $$\lim_{x\to 0}\frac{er\;f(x)}{x^{2}}=\infty \;.$$

Therefore, care is required when treating this singular term. By taking note of this fact, Equation (A24) reads:

(A26) $$I_{1}\left(a\right)=\frac{1}{2}\;\frac{e^{-x^{2}}\;er\;f\left(a\;x\right)}{x^{2}}{|}_{x\to 0}-\int_{0}^{\infty }\frac{dx}{x}\;e^{-x^{2}}\;er\;f\left(a\;x\right)+\frac{a}{\sqrt{\pi }}\;\int_{0}^{\infty }\frac{dx}{x^{2}}\;e^{-\left(a^{2}+1\right)\;x^{2}}\;.$$

One calculates the first integral in the right-hand-side of Equation (A26) from the formula:

(A27) $$\int_{0}^{\infty }dx\;e^{-x^{2}}\;\frac{er\;f(a\;x)}{x}=\sinh^{-1}\left(a\right)\;.$$

On the other hand, the second (last) integral in the right-hand-side of Equation (A26) is problematic because it has singularities. To calculate it we use the indeterminate integral formula:

(A28) $$\int \frac{dx}{x^{2}}\;e^{-a^{2}\;x^{2}}=-\frac{e^{-a^{2}\;x^{2}}}{x}-\sqrt{\pi }\;\;a\;\;er\;f\left(a\;x\right)\;.$$

Note that: aerf(ax)=|a|erf(|a|x) where $\left|a\right|=\sqrt{a^{2}}$. Thus, we proceed and rewrite Equation (A28) as:

(A29) $$\int \frac{dx}{x^{2}}\;e^{-a^{2}\;x^{2}}=-\frac{e^{-a^{2}\;x^{2}}}{x}-\sqrt{\pi }\;\;\sqrt{a^{2}}\;\;er\;f\left(\sqrt{a^{2}}\;x\right)\;.$$

By using Equation (A29), one obtains:

(A30) $$\int_{0}^{\infty }\frac{dx}{x^{2}}\;e^{-a^{2}\;x^{2}}=\frac{e^{-a^{2}\;x^{2}}}{x}{|}_{x\to 0}-\sqrt{\pi }\;\sqrt{a^{2}}\;.$$

After replacing $"a^{2}"$ with $"a^{2}+1"$ in the result of Equation (A30) one has:

(A31) $$\int_{0}^{\infty }\frac{dx}{x^{2}}\;e^{-\left(a^{2}+1\right)\;x^{2}}=\frac{e^{-\left(a^{2}+1\right)\;x^{2}}}{x}{|}_{x\to 0}-\sqrt{\pi }\;\sqrt{a^{2}+1}\;.$$

By substituting the results from Equations (A27) and (A31) into Equation (A26) one obtains:

(A32) $$I_{1}\left(a\right)=\frac{1}{2}\;\frac{e^{-x^{2}}\;er\;f\left(a\;x\right)}{x^{2}}{|}_{x\to 0}-\sinh^{-1}\left(a\right)+\frac{a}{\sqrt{\pi }}\;\frac{e^{-\left(a^{2}+1\right)\;x^{2}}}{x}{|}_{x\to 0}-a\;\sqrt{a^{2}+1}\;.$$

Let’s now calculate $I_{2}\left(a\right)$ from Equation (A23). We consider the indeterminate integral:

(A33) $$\int \frac{dx}{x^{3}}\;er\;f\left(a\;x\right)=-\frac{a}{\sqrt{\pi }}\;\frac{e^{-a^{2}\;x^{2}}}{x}-a^{2}\;erf\left(a\;x\right)-\frac{er\;f(a\;x)}{2\;x^{2}}\;.$$

With help from Equation (A33) and a careful calculation of with a careful calculation of the x→0 and x→∞ limits, one eventually has:

(A34) $$I_{2}\left(a\right)=-a\;\sqrt{a^{2}}+\frac{a}{\sqrt{\pi }}\;\frac{e^{-a^{2}\;x^{2}}}{x}{|}_{x\to 0}+\frac{er\;f(a\;x)}{2\;x^{2}}{|}_{x\to 0}\;.$$

At this point, one substitutes the results from Equations (A32) and (A34) into the expression in Equation (A21). It is easy to check that all singular terms disappear in the x→0 limit when the quantity $I\left(a\right)=I_{1}\left(a\right)-I_{2}\left(a\right)$ is calculated. The final expression of the integral can be written as:

(A35) $$I\left(a\right)=a\;\left(\sqrt{a^{2}}-\sqrt{a^{2}+1}\right)-\sinh^{-1}\left(a\right)\;.$$

The reader can easily verify that the expected result, I(−a)=−I(a).
